# Meeting Report: Measuring Endocrine-Sensitive Endpoints within the First Years of Life

**DOI:** 10.1289/ehp.11226

**Published:** 2008-04-03

**Authors:** Tye E. Arbuckle, Russ Hauser, Shanna H. Swan, Catherine S. Mao, Matthew P. Longnecker, Katharina M. Main, Robin M. Whyatt, Pauline Mendola, Melissa Legrand, Joanne Rovet, Christine Till, Mike Wade, John Jarrell, Stephen Matthews, Guy Van Vliet, Carl-Gustaf Bornehag, Roger Mieusset

**Affiliations:** 1 Biostatistics and Epidemiology Division, Health Canada, Ottawa, Ontario, Canada; 2 Department of Environmental Health, Harvard School of Public Health, Boston, Massachusetts, USA; 3 University of Rochester School of Medicine and Dentistry, Rochester, New York, USA; 4 Harbor-UCLA Medical Center, Torrance, California, USA; 5 Epidemiology Branch, National Institute of Environmental Health Sciences, National Institutes of Health, Department of Health and Human Services, Research Triangle Park, North Carolina, USA; 6 University Department of Growth and Reproduction, Rigshospitalet, Copenhagen, Denmark; 7 Columbia University Children’s Center for Environmental Health, New York, New York, USA; 8 U.S. Environmental Protection Agency, National Health and Environmental Effects Research Laboratory, Chapel Hill, North Carolina, USA; 9 Environmental Health Surveillance Division, Health Canada, Ottawa, Ontario, Canada; 10 Neuroscience and Mental Health Program and; 11 Department of Psychology, The Hospital for Sick Children, Toronto, Ontario, Canada; 12 Environmental Health Science and Research Bureau, Health Canada, Ottawa, Ontario, Canada; 13 Department of Obstetrics and Gynecology, University of Calgary, Calgary, Alberta, Canada; 14 Department of Physiology, University of Toronto, Toronto, Ontario, Canada; 15 Department of Pediatrics, University of Montreal, Montreal, Quebec, Canada; 16 Department of Mechanical Engineering, Technical University of Denmark, Lyngby, Denmark; 17 University Male Sterility Center, Toulouse, France

**Keywords:** anogenital distance, anthropometry, endocrine disruptors, genital exam, hormones, infants, measurement, neurodevelopment, reproductive tract development, sexual dimorphism

## Abstract

An international workshop titled “Assessing Endocrine-Related Endpoints within the First Years of Life” was held 30 April–1 May 2007, in Ottawa, Ontario, Canada. Representatives from a number of pregnancy cohort studies in North America and Europe presented options for measuring various endocrine-sensitive endpoints in early life and discussed issues related to performing and using those measures. The workshop focused on measuring reproductive tract developmental endpoints [e.g., anogenital distance (AGD)], endocrine status, and infant anthropometry. To the extent possible, workshop participants strove to develop or recommend standardized measurements that would allow comparisons and pooling of data across studies. The recommended outcomes include thigh fat fold, breast size, vaginal cytology, AGD, location of the testis, testicular size, and growth of the penis, with most of the discussion focusing on the genital exam. Although a number of outcome measures recommended during the genital exam have been associated with exposure to endocrine-disrupting chemicals, little is known about how predictive these effects are of later reproductive health or other chronic health conditions.

A number of pregnancy cohort studies examining the risks of potential endocrine-disrupting chemicals on pregnancy and infant and child health are currently underway in North America and Europe. Given the current interest in the reproductive and developmental effects of endocrine-modulating chemicals and ongoing discussion about the appropriate methods for measuring infant endpoints, consensus is needed on robust and validated methods that can be applied across studies so consistency of results can be evaluated or, where statistical power is limited, data can be pooled. Twenty investigators from Canada, the United States, and Europe attended the workshop “Assessing Endocrine-Related Endpoints within the First Years of Life” (30 April–1 May 2007, Ottawa, Ontario, Canada) and discussed their experiences in measuring endocrine-sensitive endpoints in the first years of life and made recommendations for future research.

The objectives of this workshop were as follows:

- To share data, experiences, and ideas for identifying and measuring postnatal endocrine-sensitive outcomes associated with exposure to environmental chemicals *in utero* and in early postnatal life, with a focus on *a*) infant reproductive tract development, *b*) markers of growth and development, and *c*) serum hormone levels
- To develop recommendations, if possible, on standardized methods for measuring indicators that would allow comparisons across studies or pooling of data among studies
- To briefly describe methods for measuring neurodevelopmental disorders and sensory dysfunction as well as prenatal and postnatal nutrition, critical cofactors in any study of endocrine-sensitive outcomes (see Supplemental Material online at http://www.ehponline.org/members/2008/11226/suppl.pdf).

## Workshop Discussion

The workshop began with invited presentations, followed by discussions of measurement issues, attempts to reach a consensus on recommended methods and identification of areas for future research. The topics and discussion were not intended to be an exhaustive survey of methods, but rather were focused on endocrine-sensitive measures where there is enough information to suggest standardization is possible and where uncertainty or debate remain about how to best make the measurement. The validity of the specific developmental endpoints discussed as indicators of a specific endocrine target in humans may still require further investigation.

### Reproductive Tract Developmental Endpoints and Anthropometric Measures

#### Logistical considerations in the conduct of the clinical exam

The timing of the exam depends on the research question, the measures to be taken, and the ability of the researchers to get the parent and infant into the clinic. In addition, researchers must be aware of developmental hormone changes that can impact measurements during early life and other sources of endocrine disruptors in the diet. Ideally, newborn clinical exams should be done within the first 24 hr after birth; however, this may result in data being collected by hospital staff members who are not fully trained, thereby increasing variability. The recommended ages for genital measurements are *a*) neonates during the first day after birth in the maternity ward, except for premature infants, where the exam should take place on the expected date of delivery; *b*) 2–4 months of age to coincide with mini-puberty, which reflects the hormonal surge of gonadotropins [predominantly luteinizing hormone (LH) in boys and follicle-stimulating hormone (FSH) in girls] that occurs in early infancy (Quigley 2002); and *c*) 6 months of age to coincide with the post-minipuberty period for most infants. (Note that girls can have elevated FSH levels up to 4 years of age.) It is recommended that clinical exams of infants take place while they are still breast-feeding (before 4–6 months of age) and before other foods are introduced, to minimize confounding by diet.

The age and developmental stage of the infant are also factors to consider when taking physical measurements during the clinical exam. Newborn measures tend to be easier to obtain, as the babies move less and are not dehydrated, and the exam is generally acceptable to the parents. Likewise, exams at 3 and 6 months and at 3 years of age tend not to be problematic. On the other hand, infants 8–9 months and 18 months of age show the most distress from being separated from their parents and may have difficulty adjusting to the new environment.

Digital photos of facial and genital features may be useful for pattern recognition, at least for discussion purposes; however, photos have not been useful for taking reliable quantitative measurements.

#### Infant girls

Genital exams of infant girls should include examination of the vulva and perineum for color intensity and moisture level, which tends to be red and glistening moist without estrogen exposure, as opposed to pink and dull if there has been increased estrogen exposure. In addition, vaginal cytology (taken with a cotton-tipped swab from the introitus) may be useful to show continued estrogen stimulation at birth and at 6 months (e.g., from soy formulas) (Bernbaum et al. 2008). The participants did not recommend measuring the clitoral index (product of the longest sagittal and transverse clitoral dimension) for research purposes. Although this index is used clinically to evaluate virilized genitalia, these measures can be inconsistent because of variation in the amount of “squeezing” force exerted by the examiner. Any labial fusion should be noted.

Qualitative measures such as labial color and rugation are not useful, as there are too few differences in prevalence and features may vary with minipuberty. Although there are a few studies of ultrasound and magnetic resonance imaging scans of the internal female genitalia, these methods may not be sensitive enough for subtle endpoint changes and require significant resources. Breast tissue size and anogenital distance (AGD) may be useful measurements.

#### Infant boys

In infant boys, the order of the genital examination is important, as repeated contact can result in some erection of the penis and retraction of the testes. The participants recommended that the exam begin with recording the testis position, followed by measures of penile width (where squeezing is less of a problem), penile length, and AGD (see Figure 1 in Romano-Riquer et al. 2007). In Denmark, calipers are used and the penis is not stretched, whereas in the United States, the penis is stretched and a disposable wooden stick is marked to record length. It is unknown which of these two approaches for measuring penile dimensions best reflects an androgen response. One disadvantage of using calipers is that they must be sterilized. Measures of penile dimensions at several time points are useful, with measures at birth representing the endocrine environment *in utero* and those at 3–6 months likely reflecting both exposures *in utero* and minipuberty.

Penis length is the accepted clinical measure of androgenization; however, penis width may also provide additional independent information. Maternal age is inversely related to stretched penis length and width in models adjusted for birth weight (Romano-Riquer et al. 2007). This finding is interesting in that older pregnant women tend to have lower testosterone levels (Carlsen et al. 2003; Troisi et al. 2003), and testosterone levels seem to be inversely related to birth size (Carlsen et al. 2006). This may also indicate that, at least in humans, the penis dimension measures may be more responsive than AGD to the intra-uterine hormone environment.

There was some discussion about whether the location of testis (Scorer 1964) should be measured quantitatively as the distance between the pubic bone and testes. Although this measurement is important, participants agreed that it was difficult to measure consistently. Some maintained that testis location should be included in any genital exam, and they noted that testicular placement is a continuum from normal (fully descended) to ectopic. Moreover, once technicians have been trained, this measure is highly repeatable. In addition, it is possible to describe the position of the testis first before manipulation and then measure how far the testis can be pulled from the pubic bone along its normal route of descent (Boisen et al. 2004). At 3 months of age, differences in the hormone profile have been observed between those infants who have a high versus low scrotal position (Suomi et al. 2006). The distance measured from pubic bone to top or middle of the testis is usually done after manipulating the testis into the lowest possible position by gentle traction. Whether or not the variation observed between infants will be substantial enough to overcome variability introduced by examiners remains to be seen and will likely be study dependent.

Several participants also noted that testis size/volume measurements may not be reliable unless ultrasound measures are available to give quantifiable volume. Extensive training is critical. There has been some success in measuring testis volume using modified Prader beads (finer gradations), but some participants felt that this measure was not useful, especially compared with the penile length and width measures. Without an ultrasound exam, it is also impossible to determine if the measurement is testis alone or includes other tissue, such as the epididymis or a small hydrocele. Given that ultrasound would add substantial cost and team effort, measures of testis size are not routinely recommended and should only be done if the specific research question requires it.

Although gradation of scrotal size (e.g., small, medium, large) can be difficult to measure, this may be a sensitive indicator of androgen effect that does vary considerably among populations. Pictures can be helpful in improving consistency, but reproducibility and scale are a concern. This measure is not routinely recommended and should be used with caution to address specific research questions.

Subjective quantification of suprapubic fat and measurement of anoscrotal distance were not recommended.

#### Anogenital distances

AGD is a measure of fetal androgen action commonly used in animal studies and recently in human studies (Longnecker et al. 2007; Swan et al. 2005). Calipers or a wooden tongue depressor that can be marked are two options for measuring genital distances. Other suggestions include dental floss or sutures. All things considered, the participants agreed that the recommended instrument for measuring AGD is a caliper for both boys and girls.

All measures should be repeated at least once. In boys, AGD is measured in various ways, including from the middle of the anus to the anterior base of the penis, from the anus to the posterior base of the penis, and from the anus to the posterior base of the scrotum. The measurement to the anterior base of the penis is easier to standardize and the most reliable. The distance can vary slightly depending on the amount of pressure exerted. In girls, AGD is measured from the center of the anus to the fourchette and from the anus to clitoris (see Figure 1 in Callegari et al. 1987).

Systematic differences between trained observers in measures of AGD have been observed; however, this variation is relatively small compared with the true differences between babies (Romano-Riquer et al. 2007). To compare different measures of AGD, the coefficient of variation may not be the best parameter to use. Instead, researchers should calculate the ratio of the true variation among subjects in the numerator and the total variation among subjects including measurement error in the denominator (inter-rater reliability).

In rodents, a small AGD at birth remains shorter in adulthood; however, it is unknown whether the same holds true for humans. It is assumed that the dynamics could change post-natally, depending on either a new exposure or, for males, a congenital testicular dysgenesis with delayed effects during pubertal and adult development. One approach would be to examine AGD in cases of ambiguous genitalia and measure changes based on hormonal status postnatally. At this point, the timing of post-natal changes is speculative, but AGD may be stable for at least the first month of life. If longitudinal data are available, the dynamics of AGD may prove more sensitive for anti-androgenic effects than a single measurement.

#### Breast tissue

The amount of breast tissue in boys and girls can be palpated, measured, and compared with fat mass (Bernbaum et al. 2008; Schmidt et al. 2002); however, breast tissue size can be difficult to distinguish from the surrounding fat, and an ultrasound may be helpful. The diameter of breast buds, if present, can be measured using disc-shaped beads in 2-mm increments from 4 to 22 mm, strung in an ordered set (Bernbaum et al. 2008). The number of nipples in humans is not sexually dimorphic; therefore, this outcome was considered to be of little importance in studies of endocrine disruption. In rodents, however, this is a useful outcome.

#### Body fat

From a research perspective, body composition and fat are important outcomes, but these are among the most challenging and potentially upsetting examinations for both the technicians and the babies. The skin fold caliper spring tends to tighten the longer it is left on, causing pinching and resulting in variability of the measure. The hardest skin fold measure to take is the thigh (quadriceps); however, the thigh is a measure with great sexual dimorphism. Because it is distressing for babies, thigh fat measures should be done at the end of the exam. Other possible measurements include circumferences of the upper arm and leg.

A bioimpedance scale can measure body composition well, but it is important to have some estimate of hydration if the measures are not taken after standardized fasting. Air-displacement plethysmography is a new technique for infant measurements that looks promising (Ellis et al. 2007).

#### Anthropometry

In addition to the measures already discussed, workshop participants recommended recording standard measures of body size (Lohman et al. 1988). Measures of birth length and head circumference noted on medical charts are not sufficiently accurate for research purposes. For birth length, the kidimeter, as used in Denmark, or an infantometer (used in North America) can accurately measure length with both legs completely extended. For weight, it is critical that the scales are routinely calibrated.

To facilitate standardization of methods for head and abdominal circumference, landmarks where the measurements will be taken must be identified. For abdominal circumference, the umbilicus can be used as a landmark. Participants felt that interpretation of waist circumference in newborns was unclear; however, this is an important postnatal measure. The recommended landmark for head circumference is a perpendicular line over the eyebrows and above the ears. Participants recommended that a paper lasso be used [such as those available through the U.K. Child Growth Foundation (2008)], which allows the technician to tighten the lasso at the largest point and allows one hand to be free.

The recommended ages for anthropometry are at birth, 3 and 6 months, 1 year, and then annually thereafter. International growth curves for children between birth and 2 years of age are available through the World Health Organization (WHO 2008).

#### Research needs

For the male infant population, observations of markers of anti-androgen or estrogenic activity are of interest; in girls, however, the impact of androgens and estrogens is less clear. For female infants, noninvasive measurements that can be obtained reliably and biomarkers for androgen and estrogen effects need to be developed.

At this point, there are no gold standard measures, with most measures having some merit. Therefore, it would be useful to compare instruments to determine which measures have the strongest association with prenatal exposures or other factors of interest. Research to help separate the impact of *in utero* exposures from the effects of the postnatal environment is needed. A related research question is how the postnatal environment can add to or modify the effects of the prenatal environment.

With respect to AGD and related measures, a major research need is identifying the best approach to normalize these by the size and age of the baby. Suggestions include adjusting for gender-specific weight percentile, age, and length.

In animal studies, AGD is an important indicator of a developmental endocrine effect; however, it is unknown whether the same is true in humans. Other research needs or data gaps include: *a*) identifying the most important cofactors that should be measured (e.g., ethnicity, anthropometry); *b*) collecting normative data; and *c*) collecting longitudinal data to examine whether relative AGD and other measures change with age.

Another unresolved issue is the utility of measuring testis volume. Significant growth in the testis occurs between birth and 3 months of age. However, growth rates can vary significantly between populations, and it is unknown whether such differences will result in any problems for the population with slower growth rates.

Research is needed on how to describe scrotal characteristics and measure its size. Pictures can be helpful in improving consistency, especially if issues of scale and approval of human subjects are adequately addressed. An unstudied area is whether, in adulthood, differences in sperm quality will be observed between those men whose testes were in a high versus low scrotal position and those whose testes were or were not retractile in infancy.

Additional research needs include better methods for measuring skin fold thickness of the thigh and additional data on inter-rater reliability of the assessments.

### Hormonal Measures

Although most hormonal measures of interest can be examined easily in blood samples, these samples are often a challenge to collect from infants and children. In Scandinavian countries, collection of blood from infants (10 mL at 3 months of age) has not been the ethical challenge that it has been in North America. Although a heel stick may be more tolerable for parents and ethics committees, it may be more painful for the infant than venipuncture (Ogawa et al. 2005). Finger pricks may be more acceptable, but less blood is obtained, and they are more painful than heel sticks.

#### Gonadal axis

The preferred age for obtaining infant blood measurements is 2–4 months of age, which represents minipuberty when peak hormone levels can be captured. For boys, this will give an indication of Leydig cell function (FSH, testosterone, and LH) and Sertoli cell function (inhibin B and FSH). Hormonal measurements in girls during minipuberty show activation of the pituitary–gonadal axis, but with a large variation within the normal range (Chellakooty et al. 2003). Minipuberty in girls can be captured by measuring FSH, estradiol, LH, and inhibin A and B. Although obtaining additional blood at the time of routinely scheduled blood draws (American Academy of Pediatrics 2007) is appealing, they generally fall outside the period of greatest interest for capturing hormone peaks.

A major problem is the poor detection limit of currently available hormone assays for estradiol and testosterone and their cross-reactivity with other hormones in the immunoassays, which is of concern especially in cord blood samples. Methods using gas chromatography/mass spectrometry are sensitive at low levels for steroid hormones.

#### Thyroid axis

To study maternal thyroid hormone levels and their effects on offspring cognitive development, maternal blood should be collected in the first trimester and analyzed for thyroid-stimulating hormone (TSH) and free thyroxine (T_4_). As part of newborn screening in most developed countries and U.S. states, TSH (and T_4_ in some places) is measured from blood collected by heel pricks spotted onto filter paper. Workshop participants recommended that thyroid hormone levels (TSH, total T4, free T_4_, and total tri-iodothyronine) be measured at 3 months of age. If infant blood cannot be collected, collecting maternal blood during the third trimester and cord blood should be considered, as this would allow the separation of maternal and fetal hormone levels (Dussault JH, Morissette J, unpublished data). Infant and maternal urine can also be analyzed for gonadotropins and iodine (which is a constituent of thyroid hormone).

The participants recommended caution when interpreting hormone levels at birth, as both the baby and mother are under trauma and stress to various extents due to delivery (unless there is a very large effect of the prenatal exposure).

Because there are a number of parallel mechanisms related to steroidogenesis, the adrenal hormones should not be ignored. Adrenal hormones can be measured in amniotic fluid, blood, and saliva, but they are difficult to measure in urine, and regardless of matrix, diurnal variation needs consideration. In saliva, only free hormones can be measured, and there is still ongoing debate as to whether saliva measures are well correlated with serum levels. Adrenocorticotrophic hormone is very pulsatile and can be measured in blood but is unlikely to be found in saliva.

#### Research needs

More data are needed on the timing of early hormonal peaks in infants. Because of the difficulty in obtaining infant blood samples, other matrices such as saliva, urine, placenta, and meconium need to be explored for measuring hormones. Collecting large volumes of saliva from infants using currently available methods can be difficult and cause abrasion with bleeding (Umbach D, Phillips T, Davis H, Archer J, Ragan B, Bernbaum J, et al., unpublished data). Filter paper may be used for collection of smaller volumes of saliva. Improved methods are needed to assay hormones on filter paper from saliva or heel stick (as part of newborn screening).

## Conclusions

Overall the participants agreed that to evaluate the utility of various measures in predicting specific health outcomes, common standardized measures have to be employed across studies. For the clinical genital exam for boys, the order of the measurements is important and should begin with recording the testis position, followed by measures of penile width, penile length, and AGD. With respect to AGD and related measures, a major research need is identifying the best approach to normalize them by the size of the baby. The recommended ages for anthropometry are at birth, 3 and 6 months, 1 year, and then annually thereafter.

The preferred age for obtaining infant blood measurements is 2–4 months of age, which represents minipuberty (Quigley 2002) when peak hormone levels can be captured. In addition to gonadal effects, other hormonal systems, such as the thyroid and adrenal glands, may also be affected by exposure to endocrine disruptors and should be included in future studies.

The fetus and young infant appear to be susceptible to endocrine-disrupting effects of environmental chemicals. As exposure during critical developmental phases such as *in utero* and in the early postnatal period may have an adverse effect on reproductive health and neurodevelopment, research in this area should be increased. Such research should continue to search for valid biomarkers of endocrine dysfunction and at the same time include the existing measurements as described above, to facilitate comparability between study groups. Preferably, such studies should be planned longitudinally to increase our knowledge on whether early markers of hormonal disturbance may have a lasting effect into puberty and adulthood.
